# Protective effects of 1*α*,25‐Dihydroxyvitamin D3 on cultured neural cells exposed to catalytic iron

**DOI:** 10.14814/phy2.12769

**Published:** 2016-06-01

**Authors:** Francesca Uberti, Vera Morsanuto, Claudio Bardelli, Claudio Molinari

**Affiliations:** ^1^ Laboratory of PhysiologyDepartment of Translational MedicineUPO – University of Eastern PiedmontNovaraItaly

**Keywords:** Active vitamin D, catalytic iron, human neuronal cells

## Abstract

Recent studies have postulated a role for vitamin D and its receptor on cerebral function, and anti‐inflammatory, immunomodulatory and neuroprotective effects have been described; vitamin D can inhibit proinflammatory cytokines and nitric oxide synthesis during various neurodegenerative insults, and may be considered as a potential drug for the treatment of these disorders. In addition, iron is crucial for neuronal development and neurotransmitter production in the brain, but its accumulation as catalytic form (Fe^3+^) impairs brain function and causes the dysregulation of iron metabolism leading to tissue damage due to the formation of toxic free radicals (ROS). This research was planned to study the role of vitamin D to prevent iron damage in neuroblastoma BE(2)M17 cells. Mechanisms involved in neurodegeneration, including cell viability, ROS production, and the most common intracellular pathways were studied. Pretreatment with calcitriol (the active form of vitamin D) reduced cellular injury induced by exposure to catalytic iron.

## Introduction

Vitamin D, particularly its active form (calcitriol, 1alpha,25‐dihydroxyvitamin D3), is known for its role in calcium and bone homeostasis, cell proliferation, and modulation of parathyroid hormone secretion (Honmou et al. 2012). Recently, a number of extraskeletal actions of vitamin D have been reported, including effects on nitric oxide production, antioxidant activity, and endothelial proliferation and migration (Molinari et al. 2011; Uberti et al. 2014; Pittarella et al. 2015). Vitamin D acts on cells and tissues that express a nuclear receptor called VDR. Although in human, most of the vitamin D that ends up in the blood is produced in the kidneys, vitamin D may be synthesized in other tissues such as endothelium, placenta, prostate, skin, colon, breast, and the central nervous system (CNS). The production of vitamin D by these tissues/organs is low compared to the amount produced by kidneys, and this vitamin D is probably not released back into the blood but acts within the tissue where it is made (Garcion et al. 2002; Tetich et al. 2004; Cranney et al. 2007). As regards the CNS, several recent studies have postulated anti‐inflammatory, immunomodulatory, and neuroprotective roles for vitamin D (Garcion et al. 2002). Both 1alpha‐hydroxylase and VDR are widely expressed in neurons and glial cells, suggesting that vitamin D may have autocrine and paracrine actions in the brain (Prüfer et al. 1999; Eyles et al. 2005). VDR is expressed in several regions (Tetich et al. 2004) of animal (Garcion et al. 2002) and human (Kalueff et al. 2006) brains, particularly in the pontine‐midbrain area, cerebellum, thalamus, hypothalamus, basal ganglia, hippocampus, olfactory system and temporal, orbital and cingulate areas of the cortex (Eyles et al. 2003). Mounting evidence indicates that vitamin D and its receptor have effects that include neuroprotection and immunomodulation (Aloia et al. 2010). Moreover, vitamin D is a regulator of brain cell proliferation and differentiation, and has an essential role in the brain development (Chopp et al. 2007; Stewart et al. 2010) and neurotransmitter synthesis (Tetich et al. 2004). Vitamin D can exert these effects due to its ability to cross the blood–brain barrier and to bind to VDR within the brain (Chowdhury et al. 2012; Kienreich et al. 2013). Upon binding, VDR forms a complex with a retinoid X receptor that controls gene expression (Arbelle et al. 1996; Thompson et al. 1998). Gestational vitamin D deficiency induces long‐lasting alterations in the brain structure, including changes in volume, cell proliferation and reduced expression of nerve growth factors, glia‐derived neurotrophic factor, and neurotrophins 3 and 4 (Eyles et al. 2003; Tetich et al. 2004; Cui et al. 2007). Moreover, vitamin D can protect neurons against NMDA, glutamate, 6‐hydroxydopamine, and reactive oxygen species (Brewer et al. 2001; Ibi et al. 2001). It has been hypothesized that vitamin D exerts these effects by modulation of neuronal Ca^2+^ homeostasis, in particular through downregulation of L‐type voltage‐sensitive Ca^2+^ channels in hippocampal neurons against excitotoxic insults (Tetich et al. 2005), accompanied by an increase in VDR density. Vitamin D can inhibit proinflammatory cytokine and nitric oxide synthesis (Garcion et al. 1997) induced during various insults or disorders, such as ischemia and reperfusion, Alzheimer's disease, Parkinson's disease, AIDS, infection, multiple sclerosis, and experimental autoimmune encephalomyelitis. For these reasons, it may be considered as a potential treatment of neurodegenerative disorders.

Early vitamin D deficiency may be a risk factor for a number of disorders including schizophrenia, autism (Thompson et al. 1998; Pilz et al. 2011; Chowdhury et al. 2012), multiple sclerosis, Parkinson's disease, and stroke (Sun et al. 2012). Several neurodegenerative disorders have been associated with the dysregulation of iron metabolism (Ponka 2004) including Huntington's disease, Parkinson's disease, and neurodegeneration with iron accumulation (Mena et al. 2015). Iron is involved in a wide range of cellular processes, including DNA synthesis and repair, energy metabolism, phospholipids metabolism, and oxidative phosphorylation (Salvador 2010; Park et al. 2015). In addition, iron is crucial for neuronal development and neurotransmitter production in the brain (Sipe et al. 2002; Park et al. 2015). However, the accumulation of iron as catalytic form (Fe^3+^) impairs the brain function, and dysregulation of iron metabolism results in tissue damage due to the formation of toxic free radicals (ROS) (Lipinski 2011; Urrutia et al. 2014; Park et al. 2015). Neurons have highly regulated mechanisms for controlling cellular iron levels. The uptake is controlled by transferrin receptor‐1 or by divalent metal transporter‐1 (DMT1; Moos et al. 2007; Dev et al. 2015). The uptaken iron is stored within ferritin to avoid iron‐mediated damage and preserved for other cellular activities (Hentze and Kuhn 1996; Rouault and Cooperman 2006; Andrews and Schmidt 2007; Dev et al. 2015). Cells also control iron levels by releasing it through ferroportin (Abboud and Haile 2000; Hentze et al. 2004; Dev et al. 2015). Inappropriate regulation of one of these mechanisms may result in accumulation of iron. Neurons cannot reduce intracellular iron by cell division, so iron concentration control depends on cellular iron homeostasis. Although iron content increases with age (Zecca et al. 2001; Gotz et al. 2004; Mura et al. 2006), a large portion of neuronal cells stay viable and control the degree of oxidative damage, presumably through the activation of adaptive mechanisms (Mura et al. 2006).

Neuronal iron accumulation is thought to be involved in several neurodegenerative disorders, but the molecular events underlying the toxicity remain unknown. Recently, a relationship between iron and vitamin D has been observed, especially in the context of iron deficiency and anemia (Lee et al. 2009). Moreover, a neuroprotective effect of vitamin D against iron‐induced oxidative injury in locus coeruleus of rat has been reported (Chen et al. 2003).

However, little is known about its role in counteracting the harmful effects of intraneuronal iron accumulation.

The present research was planned to study the effects of vitamin D on iron accumulation after 6 days of stimulation in BE(2)M17 neuroblastoma cells. (Filograna et al. 2015). Mechanisms involved in neurodegeneration, such as cell viability, ROS production, and the most common intracellular pathways were studied.

## Material and Methods

### Cell culture

Human neuroblastoma BE(2)‐M17 cells were cultured as previously described (Filograna et al. 2015) in a 1:1 mixture of Ham's F12 and Dulbecco's Modified Eagle's Medium (Sigma‐Aldrich, Milan) supplemented with 10% fetal bovine serum (FBS, Sigma‐Aldrich, Milan), 1% penicillin/Streptomycin (Sigma‐Aldrich, Milan), and grown in the presence of 5% CO2 in an incubator at 37°C. In all experiments, cells were used at early passages (P1‐5 after purchase from LGC standards). To study cell viability (MTT test) and ROS production, 1 × 10^4^ cells were plated on 96‐well plates; to perform iron staining (Perl's and Turnbull), immunocytochemistry and immunofluorescence studies, 0.25 × 10^4^ cells were placed in CultureSlide (BD, Bedford, MA) with four chambers; to analyze the intracellular pathways through western blot analysis, cells were plated on 60‐mm culture dishes until confluence.

### Experimental protocol

BE(2)M17 cells were treated with different concentrations of catalytic iron (Fe^3+^) in a range (from 75 to 300 μmol/L) that was able to induce neurodegeneration (Park et al. 2015). The same stimulations were replicated after pretreatment with calcitriol in a range (0.1–100 nmol/L) reported in literature (Bini et al. 2012) as causing no significant change in cell viability. Before stimulation, cells were synchronized overnight in serum‐free medium and incubated in the culture medium without red‐phenol supplemented with 5% FBS, 1% penicillin/streptomycin, before and during treatment. Stimulation with 1 nM calcitriol was maintained for 6 days and replicated as pretreatment for 6 days before stimulation with catalytic iron for another 6 days.

### Iron staining

Perls’ stain was used to visualize, respectively, ferric iron (Fe^3+^) and ferrous iron (Fe^2+^) in BE(2)M17 cells as reported in the literature (Meguro et al. 2007; Dang et al. 2011; Owen et al. 2016). Cells cultured in chamber slide, as described before, were washed three times with cold PBS 1× supplemented with 2 mmol/L sodium orthovanadate, and fixed using a cold fixative solution (3.7% formaldehyde, 3% sucrose in PBS 1×) for 20 min at RT. Slides to be stained for Fe^3+^ were incubated with potassium ferrocyanide, then with potassium ferrocyanide + HCl, for 30 min each (Perls’ method). Finally, they were counterstained with safranin‐O and mounted with Bio Mount (Bio‐Otika, Milan, Italy). The intensity of Perls’ staining was semiquantified from five photomicrographs per chamber (Owen et al. 2016). All micrographs were taken at the same magnification. Luminosity signals were determined using ImagePro 3 software (NIH, Bethesda, US), and the results were expressed as means ± SD (%).

### MTT test

MTT dye (Sigma‐Aldrich) was used to determine cell viability. After stimulation, cells were incubated with 1% MTT dye for 2 h at 37°C in an incubator as previously described (Uberti et al. 2014; Lattuada et al. 2015). The purple formazan crystals (3‐[4,5‐dimethylthiazol‐2‐y1]‐2,5‐ diphenyltetrazoliumbromide) were dissolved in DMSO. Cell viability was determined by measuring the absorbance through a spectrometer (VICTORX3 Multilabel Plate Reader) at 570 nm with correction at 690 nm, and cell viability calculated by comparing results to control cells (100% viable).

### ROS production

The rate of superoxide anion release was used to examine the effects of calcitriol against the oxidative stress induced by catalytic iron. Superoxide anion production was measured as superoxide dismutase‐inhibitable reduction of cytochrome C, as previously described (Uberti et al. 2014). In all samples (both stimulated and untreated), 100 *μ*L of cytochrome C was added, and in one group, 100 *μ*L of superoxide dismutase was also added for 30 min in the incubator (all substances from Sigma‐Aldrich). The absorbance changes in the sample supernatants were measured at 550 nm in a spectrometer (VICTORX3 Multilabel Plate Reader). O2 was expressed as nanomoles per reduced cytochrome C per microgram of protein, using an extinction coefficient of 21,000 mL/cm, after interference absorbance subtraction (Lattuada et al. 2015).

### Western Blot for ferritin, VDR, p53, c‐Myc, Ki67, and ERK^1/2^/MAPK

Cells were washed and then lysed in ice with Complete Tablet buffer (Roche) supplemented with 2 mmol/L sodium orthovanadate, and 1:1000 phenylmethanesulfonylfluoride (PMSF; Sigma‐Aldrich). A quantity of 35 *μ*g of protein from each lysate was loaded on 15 or 5% SDS‐PAGE gels and transferred to polyvinylidene fluoride membranes (PVDF, GE Healthcare Europe GmbH, Milan, Italy). They were incubated overnight at 4°C with specific primary antibody: anti‐ferritin (1:250, Santa‐Cruz), anti‐VDR (D‐6) (1:200, Santa‐Cruz), anti‐p53 (1:500, Santa‐Cruz), anti‐cMyc (1:200, Millipore S.p.A., Milan, Italy), anti‐Ki67 (1:500, Santa‐Cruz), anti‐Phospho‐p44/42 MAPK (Thr202/Tyr204, 1:1000 Cell‐Signaling). Protein expression was normalized and verified through ß‐actin detection (1:5000; Sigma, Milan, Italy).

### c‐Myc, Ki67 immunocytochemistry

Cells, fixed as described above, were washed twice with cold PBS 1X, permeabilized with cold PBS 1× with cold 0.5% Triton X‐100 on ice at 4°C for 20 min and washed with PBS 1×. Then, the chamber slides were incubated with 3% hydrogen peroxide in PBS 1× for 8 min to block endogenous peroxidase activity and maintained in a blocking solution composed of PBS 1× with 3% albumin from bovine serum (BSA, Sigma, Milan, Italy) for 1 h at room temperature. The slides were incubated overnight at 4°C with specific primary antibody: 1:50 c‐Myc, 1:50 Ki67. All antibodies were diluted in PBS 1× in a humidified chamber and then incubated for 20 min with diluted biotinylated secondary antibody solution (Dako Italia, Milan, Italy) and then for 20 min with VECTASTAIN^®^ ABC Reagent (Dako Italia, Milan, Italy). Finally, sections were washed, incubated with peroxidase substrate solution until the desired stain intensity had developed (Peroxidase/DAB, Dako Italia, Milan, Italy), rinsed in tap water, counterstained with Mayer's hematoxylin, and mounted with Bio Mount (Bio‐Optica, Milan, Italy). The number of positive cells was calculated as described elsewhere (Lee et al. 2009); briefly, 12 different areas (1 mm^2^) randomly selected from each section were taken, and the number of signals was determined using ImagePro 3 software (NIH, Bethesda, US). The results were expressed as a means ± SD (%).

### p53 immunofluorescence

Cells, fixed as described above, were washed with cold PBS 1X, permeabilized with cold PBS 1× with 0.5% Triton X‐100 for 20 min at 4°C, incubated in blocking solution (1% BSA and 5% FBS in PBS 1X) for 30 min at RT and then treated with p53 specific antibody (1:50, Santa‐ Cruz) in PBS 1× overnight at 4°C. The slides were then incubated with fitch‐secondary antibodies (1:200, Sigma‐Aldrich) in PBS 1× for 1 h in the dark, counterstained with DAPI (1 *μ*g/mL; Sigma‐ Aldrich) diluted in PBS 1× for 5 min in the dark at room temperature and finally mounted in Vectashield (D.B.A. Italia). The number of positive cells was calculated as described by Lee et al. (2009): briefly, 12 different areas (1 mm^2^) randomly selected from each section were taken, and the number of signals was determined using ImagePro 3 software (NIH, Bethesda, US). The results were expressed as means ± SD (%).

### Statistical analysis

For each experimental protocol, at least four independent experiments were run; the results are expressed as means ± SD of four technical replicates. One‐way ANOVA followed by Bonferroni post hoc test was used for statistical analysis, and pairwise differences compared by Mann‐Whitney U tests. *P*‐values <0.05 were considered statistically significant.

## Results

### Cell viability and reactive oxygen species (ROS) production in neuroblastoma cells

**Figure 1:**
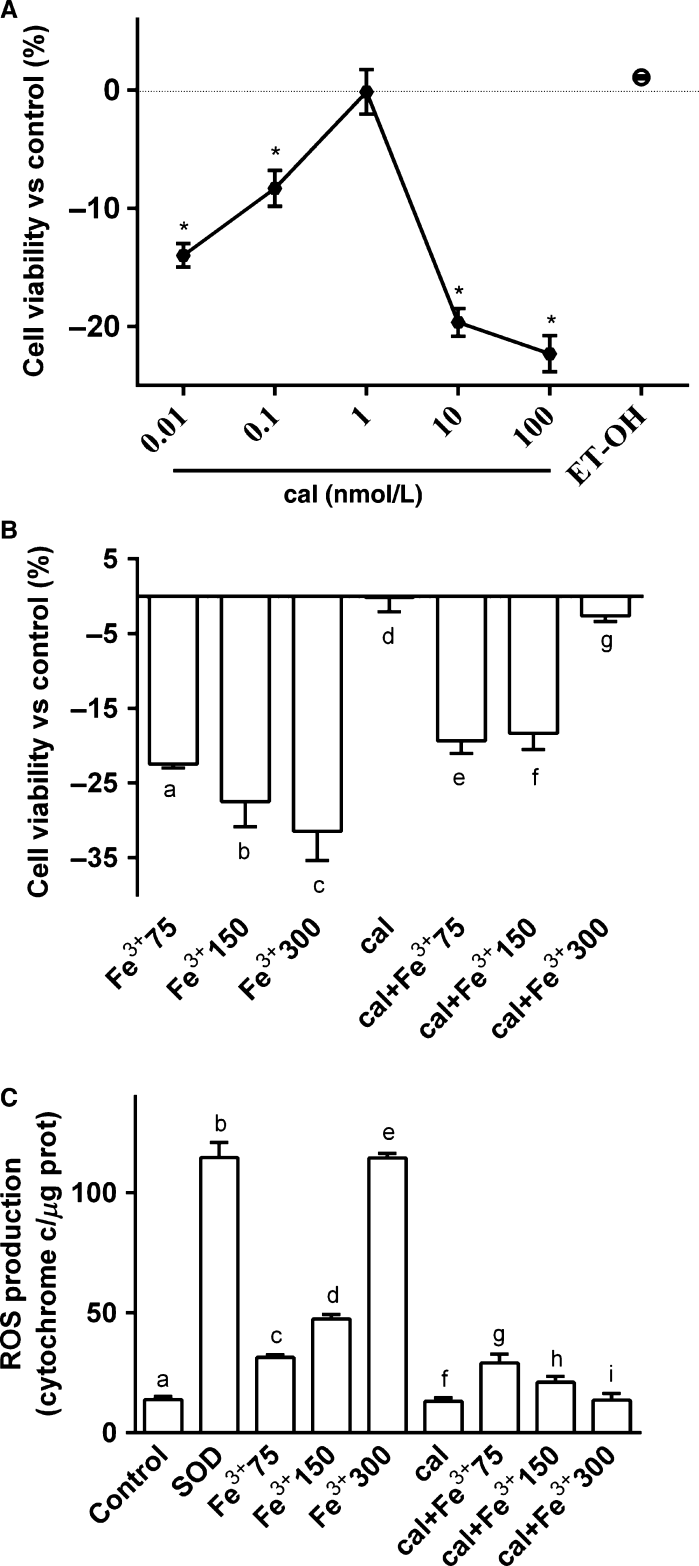
Effects of calcitriol (cal) on cell viability and ROS production. (A) dose–response study of vitamin D (0.01–100 nmol/L) on viability of BE(2)M17 cells. cal = calcitriol; ET‐OH = ethanol. *P < 0.05 versus control. (B) MTT test in cells treated with catalytic iron (75–300 μmol/L) alone for 6 days or pretreated with 1 nmol/L calcitriol for 6 days. a, b, c, e, f versus control (0%); b, c, e *P *<* *0.05 versus a; a, c, f *P *<* *0.05 versus b; a, b, c *P *<* *0.05 versus c; e, f, g *P *<* *0.05 versus d; e, f *P *<* *0.05 versus g. (C) ROS production (expressed as cytochrome C reduced per *μ*g of protein) in cells treated with catalytic iron (75–300 μmol/L) alone for 6 days or pretreated with 1 nmol/L calcitriol for 6 days. b, c, d, e, g *P *<* *0.05 versus a; g, h *P *<* *0.05 versus f; g *P *<* *0.05 versus h; c, d *P *<* *0.05 versus e; c *P *<* *0.05 versus d. In both panels, results are expressed as means ± SD (%) of four technical replicates normalized to control values.

The effect of calcitriol was analyzed by the stimulation of BE(2)M17 cultures with different doses (from 0.01 to 100 nmol/L, dissolved in ethanol) measuring cell viability through MTT test after 6 days. As illustrated in a dose–response study (Fig. 1A), after 6 days, the effect of calcitriol was concentration‐dependent, with a minimally effective dose of 1 nmol/L (−0.17 ± 1.89% vs. control). This concentration was used for all successive experiments. The effect of the solvent of calcitriol was also tested.

The influence of calcitriol on the effects of Fe^3+^ on the viability of BE(2)M17 cells was studied. As shown in Figure 1B, catalytic iron alone decreased cell viability in a concentration and time‐dependent manner, confirming data reported previously (Park et al. 2015). A maximal reduction was observed in the presence of 300 μmol/L Fe^3+^ (−31.5 ± 3.9% vs. control, *P* < 0.05). Pretreatment with calcitriol counteracted the decrease on cell viability induced by catalytic iron in a dose‐dependent manner and the maximum effect, of about 90% was observed in the presence of 300 μmol/L calcitriol (*P* < 0.05 vs. catalytic iron alone).

The same conditions were reproduced to analyze ROS production in BE(2)M17 cells (Fig. 1C). In cells treated with calcitriol alone, no significant release of ROS into the cell medium was observed demonstrating that the treatment did not lead to cell death. On the contrary, in cells treated with catalytic iron alone, there was a significant dose‐dependent increase in ROS production (Fig. 1C, *P* < 0.05). The maximum effect was obtained by 300 μmol/L (114.6 ± 1.84 of cytochrome C reduced per *μ*g of protein). Pretreatment with calcitriol was able to counteract the ROS production induced by catalytic iron and this effect was more evident in the presence of 150 μmol/L calcitriol (21.1 ± 2.5 cytochrome C reduced per *μ*g of protein; *P* < 0.05) and 300 μmol/L Fe^3+^ (13.6 ± 2.8 cytochrome C reduced per *μ*g of protein; *P* < 0.05) compared to catalytic iron alone (47.4 ± 1.86 and 114.6 ± 1.84 cytochrome C reduced per *μ*g of protein, respectively; *P* < 0.05). These data demonstrate the ability of calcitriol to prevent the negative effects of oxidative stress caused by catalytic iron on neural cells.

### Analysis of iron accumulation after 6 days of treatment with catalytic iron

**Figure 2:**
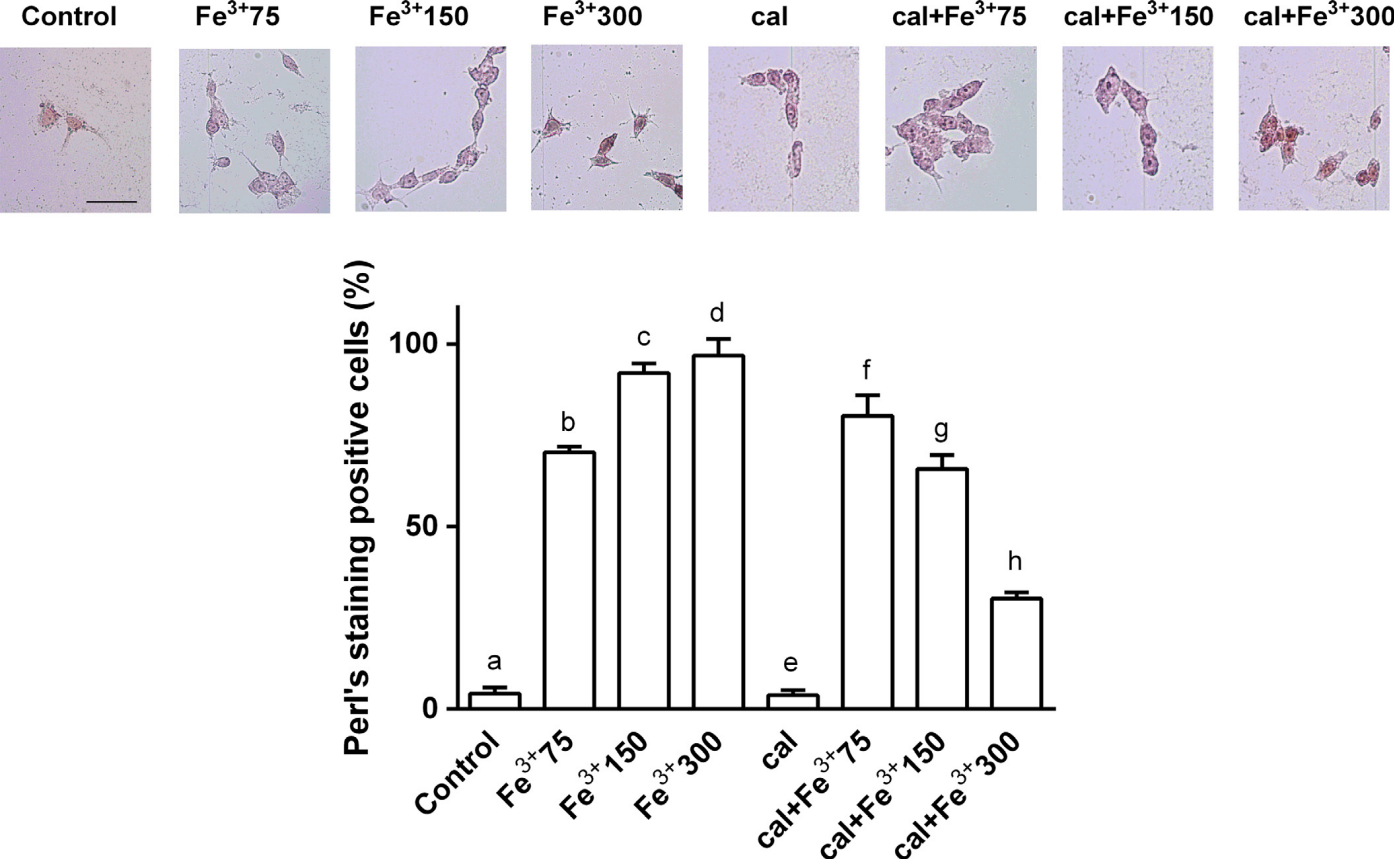
Perl's staining to visualize ferric iron accumulation after pretreatment for 6 days with 1 nmol/L calcitriol (cal) and treatment with Fe^3+^ (from 75 to 300 μmol/L) for 6 days on BE(2)M17 cells. Representative pictures reported were an example of four technical replicates taken by microscopy at original magnification ×40. The scale bar (50 μmol/L) in the first image is valid for all pictures. The ratio reported is a mean (±SD) (%) of positive cells counted in 12 different areas. b, c, d, f, g, h *P *<* *0.05 versus a; c, d, f *P *<* *0.05 versus b; h *P *<* *0.05 versus d; f, g, h *P *<* *0.05 versus e; f, g *P *<* *0.05 versus h; g *P *<* *0.05 versus h.

Perls's stain for ferric iron (catalytic) was used to study the involvement of vitamin D in preventing iron accumulation. Calcitriol alone did not influence Perls's stain compared to control (*P* > 0.05; Fig. 2). On the contrary, in neuroblastoma cells treated with different concentrations of catalytic iron, a dose‐dependent accumulation of Fe^3+^ was observed. The maximum effect was observed in the presence of 300 μmol/L Fe^3+^ and the highest concentration was observed in the perinuclear regions of living cells. Pretreatment with calcitriol was able to modulate this accumulation. Indeed, the number of positive cells significantly decreased (of about 65%; *P* < 0.05) compared to catalytic iron treatment alone.

**Figure 3:**
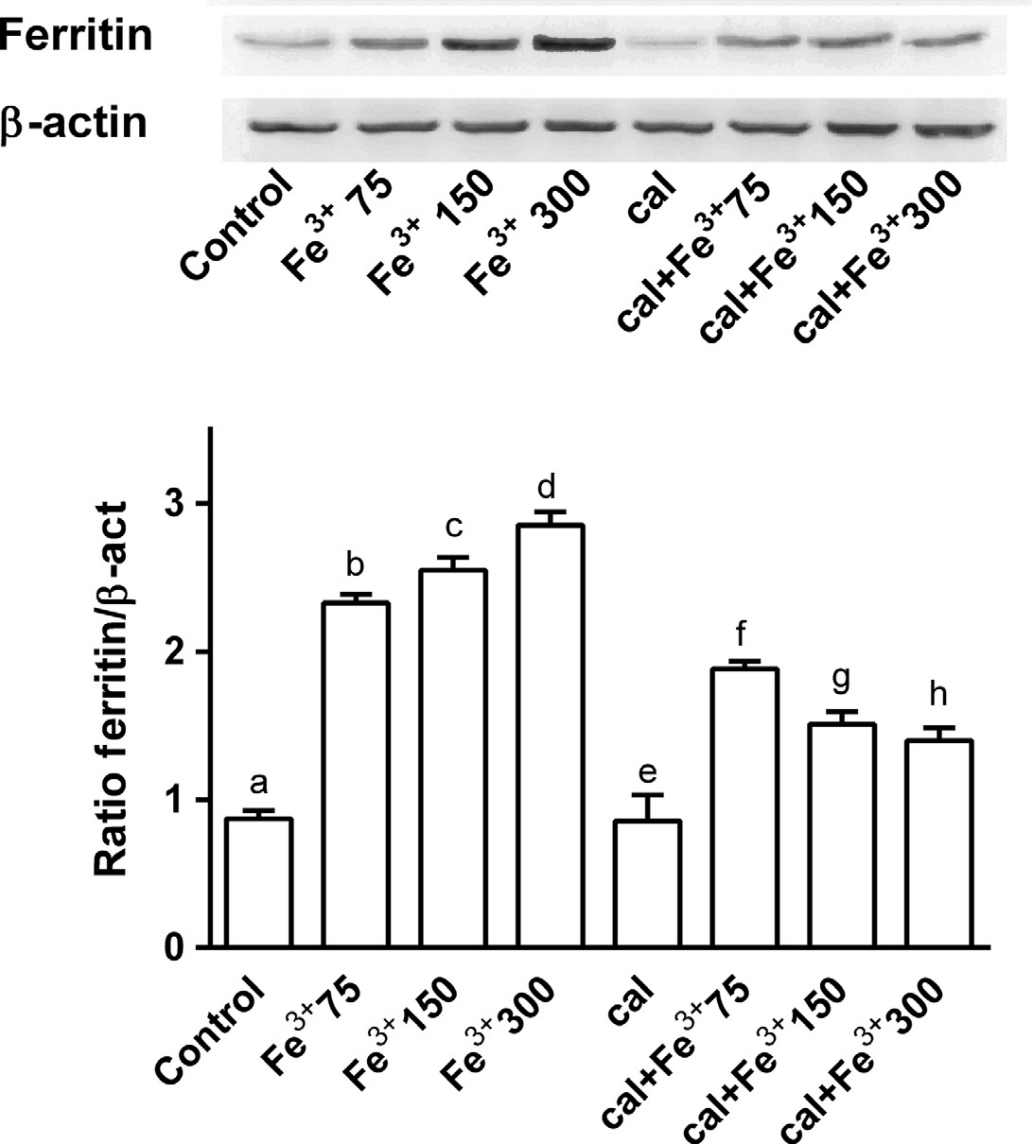
Ferritin studies by western blot and densitometric analysis after pretreatment of BE(2)M17 cells for 6 days with 1 nmol/L calcitriol (cal) and treatment with Fe^3+^ (from 75 to 300 μmol/L) for 6 days. Protein extracts were analyzed by immunoblotting with specific antibodies against the indicated proteins; results are expressed as means ± SD (%) normalized to control values of four technical replicates; b, c, d, f, g, h *P *<* *0.05 versus a; f *P *<* *0.05 versus b; d, g *P *<* *0.05 versus c; f, g, h *P *<* *0.05 versus e; g *P *<* *0.05 versus f.

To confirm these data, ferritin, a protein involved in iron storage, was measured by western blot (Fig. 3). Ferritin, the main iron storage protein in mammalian cells, is considered to be the first line of defense against iron overload. Exposure to increasing concentrations of catalytic iron from 75 to 300 μmol/L caused a significant increase in cell ferritin content (Fig. 3). At 300 μmol/L extracellular Fe^3+^, ferritin showed a tripled increase (*P* < 0.05 vs. control). On a molar basis, ferritin produced a greater increase than catalytic iron. Pretreatment with calcitriol prevented this increase in ferritin expression: at 300 μmol/L, ferritin doubled (*P* < 0.05) compared to control. These data confirmed the observations described in Figure 2 on the protective effects of calcitriol against damage depending on iron accumulation.

### Study of the intracellular pathways activated by calcitriol

**Figure 4:**
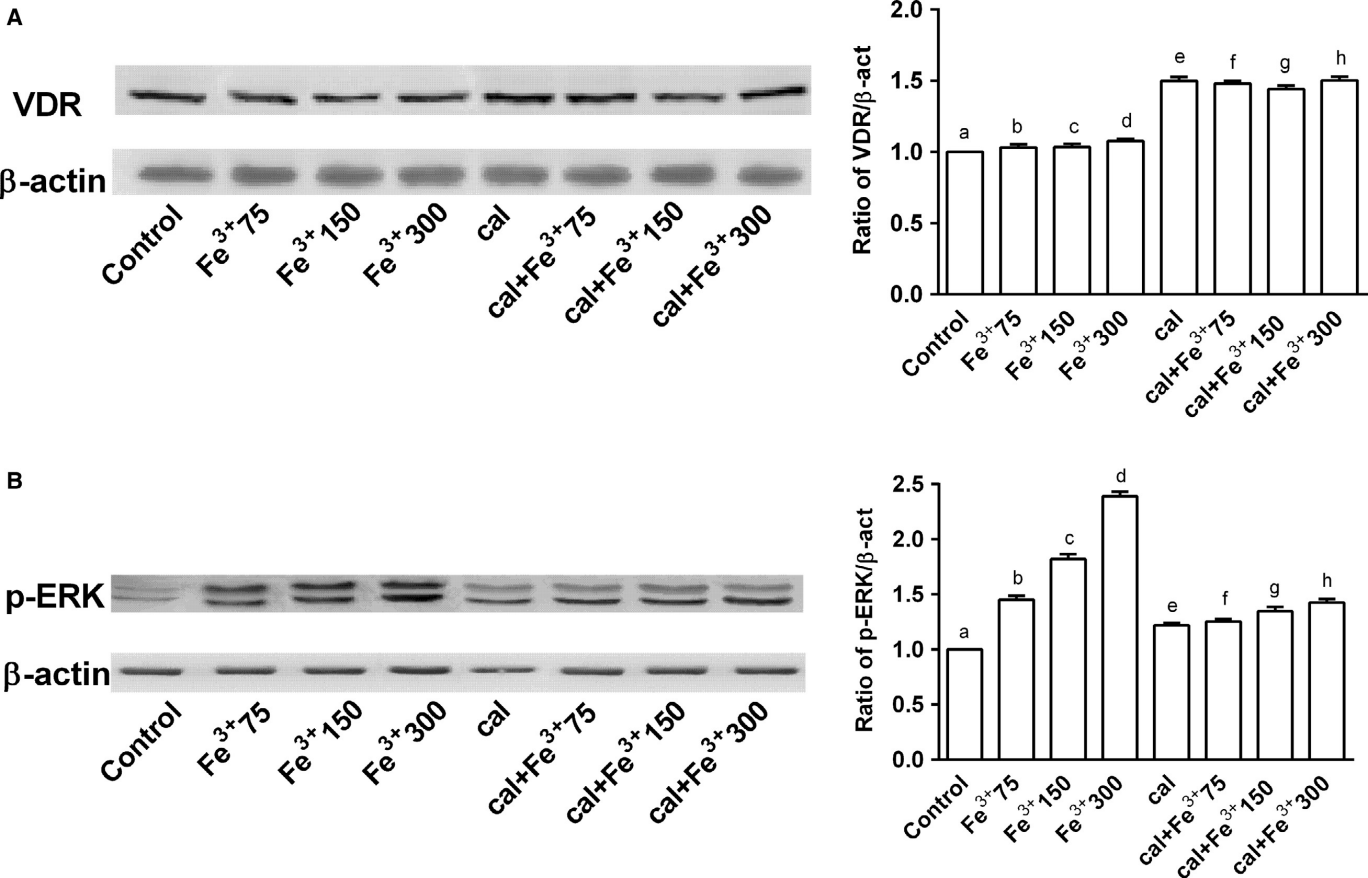
Western blot and densitometric analysis after pretreatment of BE(2)M17 cells for 6 days with 1 nmol/L calcitriol (cal) and treatment with catalytic iron (75–300 μmol/L) for 6 days. Results are expressed as means ± SD (%) normalized to control values of four technical replicates. (A) shows western blot of protein extracts analyzed by immunoblotting with a specific antibody against VDR and densitometric analysis of VDR. d, e, f, g, h *P *<* *0.05 versus a; f *P *<* *0.05 versus b; g *P *<* *0.05 versus e; f, h *P *<* *0.05 versus g; h *P *<* *0.05 versus d; g *P *<* *0.05 versus c. (B) shows western blot of protein extracts analyzed by immunoblotting with a specific antibody against p‐ERK and densitometric analysis of p‐ERK. b, c, d, e, d, f, g, h *P *<* *0.05 versus a; c, d, f *P *<* *0.05 versus b; d, g, *P *<* *0.05 versus c; g, h *P *<* *0.05 versus e; g, h *P *<* *0.05 versus f; h *P *<* *0.05 versus g; h *P *<* *0.05 versus d.

The presence of VDR receptors in neuroblastoma cells was studied by western blot and densitometric analysis (Fig. 4A). The protein expression was augmented compared to control (about 50%, *P* < 0.05) in the presence of calcitriol alone, demonstrating that calcitriol may exert its effects by VDR receptor signaling. The samples treated with Fe^3+^ did not induce a significant change in VDR expression, but in samples pretreated with calcitriol, an increase in VDR expression was observed compared to control, indicating the possible involvement of VDR in the protective effects exerted by calcitriol. In this context, the role of MAPK, a marker of cell proliferation, was examined. In samples treated with calcitriol alone, the activation of ERK was similar to control, confirming data obtained from MTT test on the ineffective dose of calcitriol (Fig. 4B). In samples treated with catalytic iron alone, the activation of ERK was overexpressed in a dose‐dependent manner, suggesting that iron‐dependent generation of ROS activates the ERK/MAPK pathway. On the other hand, pretreatment with calcitriol attenuated the overexpression caused by catalytic iron (*P* < 0.05).

### Analysis of the neurodegenerative markers: p‐53, Ki67, c‐myc

**Figure 5:**
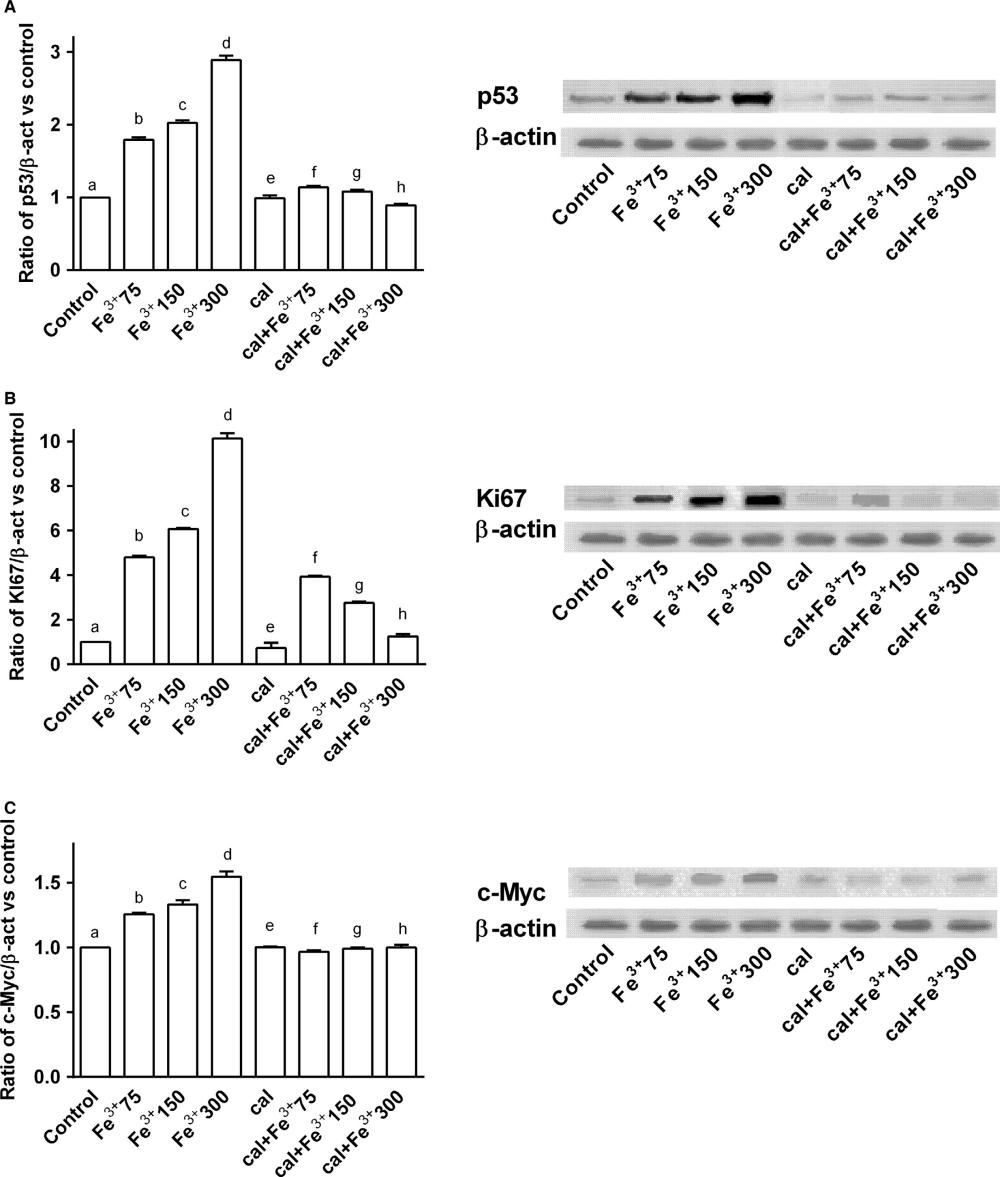
Western blot and densitometric analysis after pretreatment of BE(2)M17 cells for 6 days with 1 nmol/L calcitriol (cal) and treatment with catalytic iron (75–300 μmol/L) for 6 days. The results are expressed as means ± SD (%) normalized to control values of four technical replicates (A) shows western blot of protein extracts analyzed by immunoblotting with a specific antibody against p53 and densitometric analysis of p53. b, c, d, f, h *P *<* *0.05 versus a; c, d, f *P *<* *0.05 versus b; d, g *P *<* *0.05 versus c; h *P *<* *0.05 versus d; f, g, h *P *<* *0.05 versus e; g, h *P *<* *0.05 versus f; h *P *<* *0.05 versus g. (B) shows western blot of protein extracts analyzed by immunoblotting with a specific antibody against ki67 and densitometric analysis of ki67. b, c, d, f, g, h *P *<* *0.05 versus a; f, g, h *P *<* *0.05 versus e; c, d, f *P *<* *0.05 versus b; d, g *P *<* *0.05 versus c; h *P *<* *0.05 versus d; g, h *P *<* *0.05 versus f; h *P *<* *0.05 versus g. (C) shows western blot of protein extracts analyzed by immunoblotting with a specific antibody against c‐Myc and densitometric analysis of c‐Myc. b, c, d *P *<* *0.05 versus a; c, d, f *P *<* *0.05 versus b; d, g *P *<* *0.05 versus c; h *P *<* *0.05 versus d; f *P *<* *0.05 versus e.

**Figure 6:**
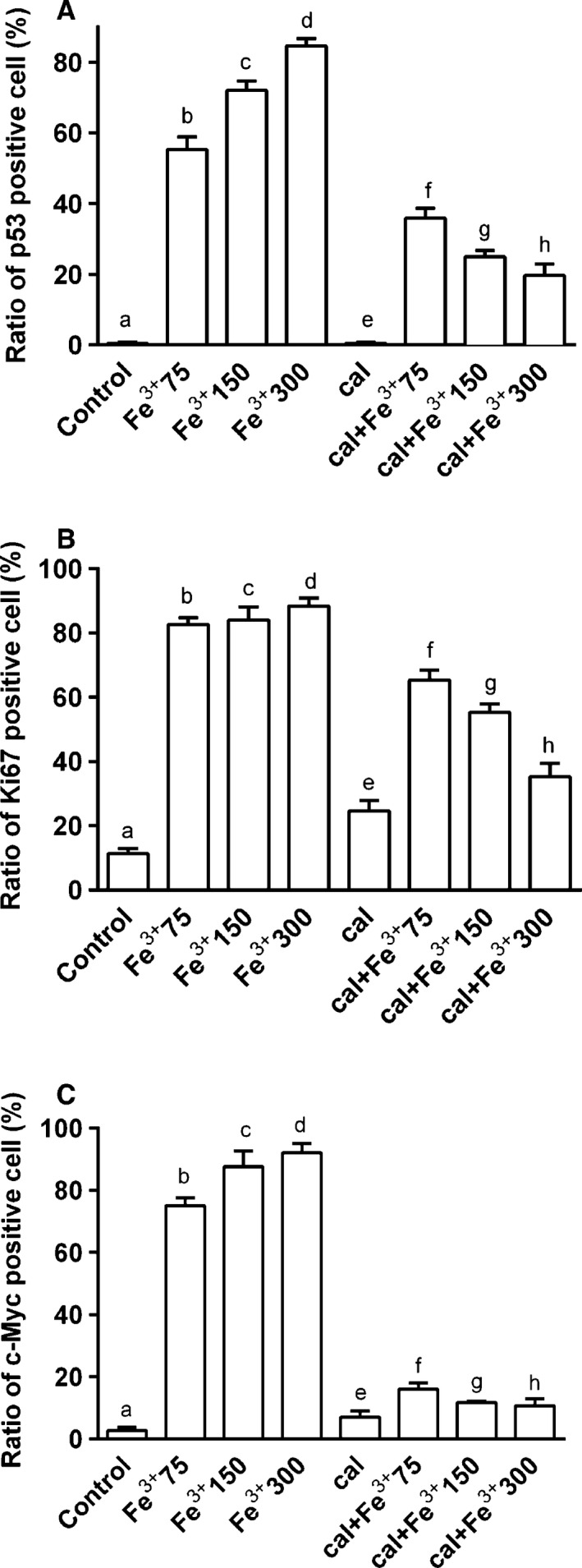
Analysis of positivity of cells by immunofluorescence and immunocytochemistry in BE(2)M17 cells pretreated for 6 days with 1 nmol/L calcitriol (cal) and treated with catalytic iron (75–300 μmol/L) for 6 days. Data are expressed as means (±SD) of positive cells counted in 12 different areas in percentage obtained by four technical replicates. (A) shows p53 positive cells. b, c, d, f, g, h *P *<* *0.05 versus a; c, d, f *P *<* *0.05 versus b; d, g *P *<* *0.05 versus c; h *P *<* *0.05 versus d; f, g, h *P *<* *0.05 versus e; g, h *P *<* *0.05 versus f; h *P *<* *0.05 versus g. (B) shows the ratio of ki67 positive cells. b, c, d, e, f, g, h *P *<* *0.05 versus a; f *P *<* *0.05 versus b; g *P *<* *0.05 versus c; h *P *<* *0.05 versus d; f, g, h *P *<* *0.05 versus e; g, h *P *<* *0.05 versus f; h *P *<* *0.05 versus g. (C) shows the ratio of c‐Myc positive cells. b, c, d, f, g, h *P *<* *0.05 versus a; c, d, f, *P *<* *0.05 versus b; g *P *<* *0.05 versus c; h *P *<* *0.05 versus d; f, g *P *<* *0.05 versus e; g, h *P *<* *0.05 versus f.

p53, Ki67, and c‐Myc expressions were investigated by western blot and densitometric analysis in BE(2)M17 cells treated with the same agents and under the same conditions as previously reported. Calcitriol alone had no significant effect on these markers (Fig. 5). In the presence of catalytic iron, dose‐dependent activations p53, Ki67, and c‐Myc were observed (Fig. 5), and maximum effects were observed with 300 μmol/L Fe^3+^ after 6 days of treatment (3, 10, and 1.5 fold activation, respectively, vs. control values). Similar data were observed in immunofluorescence (p53) and immunocytochemistry experiments (Ki67, c‐Myc; Fig. 6). Pretreatment with calcitriol for 6 days counteracted the iron‐induced activation of p53, Ki67, and c‐Myc (Figs 5 and 6). These effects were more evident in samples treated with 300 μmol/L Fe^3+^ (0.9, 1, and 0 fold of activation, respectively).

## Discussion

Although iron is an essential element, its intraneuronal accumulation can be toxic due to its capability of switching between ferrous and ferric states. The latter impairs iron metabolism, leading to neurodegenerative disorders including Alzheimer's and Parkinson's diseases (Smith et al. 1997; Takanashi et al. 2001).

As regards the mechanism of iron‐induced neuronal damage, it has been hypothesized that iron acquired by neurons accumulates in mitochondria. Neuronal death is preceded by the change in the mitochondria morphology, from a continuous to a fragmented profile, and by the loss of the mitochondrial membrane potential (Pelizzoni et al. 2011; Park et al. 2015). Another important factor implicated in mitochondrial fragmentation is the oxidative stress, also associated with several neurodegenerative disorders (Urrutia et al. 2013; Singh et al. 2014; Dev et al. 2015). Iron‐associated oxidative stress is due to its participation in the Fenton reaction, which generates ROS and consequently induces irreversible damage to DNA, RNA, proteins, and lipids (Urrutia et al. 2014). In this context, a relationship between iron, oxidative stress, and neuronal loss has been established (Gao et al. 2009; Salvador and Oteiza 2011). To reduce iron damage in the brain, Chen et al. (2003) first suggested a possible use of vitamin D supplementation, after reporting that systemic administration of vitamin D attenuates iron‐induced oxidative injuries in the rat locus coeruleus. Thus, there is evidence that the neuroprotective action of vitamin D involves neuronal calcium regulation, antioxidative pathway, immunomodulation, and detoxification.

This study demonstrates that calcitriol can prevent Fe^3+^‐dependent oxidative injury in a neuronal cell line. The beneficial effect consists in decrease in iron accumulation and ROS production, two co‐factors of neurodegeneration (Cantu et al. 2009). In addition, the observed effects of calcitriol in BE(2)M17 cells involved the activation of VDR receptor. Moreover, under our conditions, the survival pathway (ERKs) in the presence of calcitriol was also activated. In addition, Fe^3+^‐dependent activation of the neurodegenerative biomarkers p53 (Herold et al. 2015), Ki67 (Wharton et al. 2005) and c‐Myc (Sleiman et al. 2011), was confirmed.

In conclusion, the results described in this work highlight that calcitriol can protect cultured BE(2)M17 cells against Fe^3+^‐related oxidative stress and iron accumulation. The results support the potential use of vitamin D supplementation as a therapeutic strategy in neurodegeneration disorders.

## Conflict of Interest

None declared.
